# 

**Published:** 1883-07

**Authors:** 


					﻿Vol. III.
'JULY, .1883-
NO. 7.-
THE
pOMtEOPATHlC pHY^lClAfl,
A MONTHLY JOURNAL OF MEDICAL SCIENCE.
Magna est veritas, et prcevalebit.’’
HL CT. LEE, LA. Z>.,
EDITOR.
CONTENTS:
(See inside of Cover.)
PHILADELPHIA:
No. 2109 CHESTNUT STREET.
UST TS "W YORK:
A. L. CHATTERTON, PUBLISHING COMPANY, Publisher’s Agents.
LXD N'DON:
ALFRED HEATH & CO., Sole Agents for Great Britain,
114 Ebury Street, Eaton Square.
Yearly Subscription, $2.50, invariably in advance.
Entered at the Philadelphia Post-Office as Second Class Mall Matter.
				

